# Targeting the Endocannabinoidome: A Novel Approach to Managing Extraintestinal Complications in Inflammatory Bowel Disease

**DOI:** 10.3390/ph18040478

**Published:** 2025-03-27

**Authors:** Dinesh Thapa, Anjali Ghimire, Leon N. Warne, Rodrigo Carlessi

**Affiliations:** 1 Curtin Medical Research Institute, Curtin Medical School, Curtin University, Perth, WA 6102, Australia; anjali.ghimire@curtin.edu.au (A.G.); leon.warne@anaesthesia.vet (L.N.W.); 2 The Vet Pharmacist, East Fremantle, WA 6158, Australia; 3 Harry Perkins Institute of Medical Research, QEII Medical Centre and Centre for Medical Research, The University of Western Australia, Nedlands, WA 6009, Australia

**Keywords:** inflammatory bowel disease, extraintestinal manifestations, endocannabinoidome, endocannabinoids, cannabinoids, metabolic disorders, microbiome

## Abstract

**Background**: Inflammatory bowel disease (IBD) is a chronic inflammatory disorder marked by persistent gastrointestinal inflammation and a spectrum of systemic effects, including extraintestinal manifestations (EIMs) that impact the joints, skin, liver, and eyes. Conventional therapies primarily target intestinal inflammation, yet they frequently fail to ameliorate these systemic complications. Recent investigations have highlighted the complex interplay among the immune system, gut, and nervous system in IBD pathogenesis, thereby underscoring the need for innovative therapeutic approaches. **Methods**: We conducted a comprehensive literature search using databases such as PubMed, Scopus, Web of Science, Science Direct, and Google Scholar. Keywords including “cannabinoids”, “endocannabinoid system”, “endocannabinoidome”, “inflammatory bowel disease”, and “extraintestinal manifestations” were used to identify peer-reviewed original research and review articles that explore the role of the endocannabinoidome (eCBome) in IBD. **Results**: Emerging evidence suggests that eCBome—a network comprising lipid mediators, receptors (e.g., CB1, CB2, GPR55, GPR35, PPARα, TRPV1), and metabolic enzymes—plays a critical role in modulating immune responses, maintaining gut barrier integrity, and regulating systemic inflammation. Targeting eCBome not only improves intestinal inflammation but also appears to mitigate metabolic, neurological, and extraintestinal complications such as arthritis, liver dysfunction, and dermatological disorders. **Conclusions**: Modulation of eCBome represents a promising strategy for comprehensive IBD management by addressing both local and systemic disease components. These findings advocate for further mechanistic studies to develop targeted interventions that leverage eCBome as a novel therapeutic avenue in IBD.

## 1. Introduction

Inflammatory bowel disease (IBD), including ulcerative colitis and Crohn’s disease, affects millions of people with rising prevalence globally [1]. While the exact etiology of IBD is unclear, it is reported to be driven by intricate crosstalk between genetic predisposition, environmental triggers, gut microbial imbalances, and immune dysfunction [2,3,4,5,6]. Despite advancements in IBD therapy, significant gaps remain, including the management of extraintestinal manifestations (EIMs), metabolic dysfunction, and quality of life concerns. Although the primary symptoms are localized in the gut, IBD often exerts systemic effects, manifesting in a variety of extraintestinal complications such as arthritis, uveitis, osteoporosis, liver disorders, skin disorders, anxiety, and depression [7,8]. These EIMs are conditions occurring outside the gastrointestinal tract yet are closely linked to IBD, significantly adding to the disease burden and impacting patients’ quality of life. They may emerge alongside active intestinal symptoms or develop independently, and in some cases, precede the onset of IBD diagnosis. The presence of EIMs not only complicates disease management but also leads to increased healthcare demands [9,10]. As novel therapeutic strategies continue to be investigated, the endocannabinoidome (eCBome) emerges as a multifaceted therapeutic target for addressing both intestinal symptoms and extraintestinal manifestations of IBD [11,12,13].

The endocannabinoidome is an expanded network of bioactive lipids, receptors, and metabolic enzymes that regulate diverse physiological functions [13]. Core components of eCBome include the canonical cannabinoid receptors CB1 and CB2, key endocannabinoids (eCBs) such as anandamide (AEA) and 2-arachidonoylglycerol (2-AG), and their metabolizing enzymes, fatty acid amide hydrolase (FAAH) and monoacylglycerol lipase (MAGL) [14,15]. CB1 receptors are primarily expressed in the central nervous system and gut epithelium, while CB2 receptors are predominant in immune cells [16,17,18,19]. Endocannabinoids are bioactive lipids that are synthesized on demand by the host in response to physiological stimuli such as neuronal activity, stress, or immune signaling. Factors such as stress, infections, inflammation, and dietary intake can influence endocannabinoid tone and signaling, thereby affecting physiological and immunological functions. For example, acute stress can transiently elevate endocannabinoid levels to restore homeostasis, while chronic stress may lead to dysregulation, contributing to pathological conditions [20]. Beyond the classical endocannabinoids, eCBome includes lipid mediators such as oleoylethanolamide (OEA) and palmitoylethanolamide (PEA), which play significant roles in modulating immune responses, maintaining gut barrier integrity, influencing gastrointestinal motility, and regulating energy homeostasis. For instance, OEA was shown to reduce intestinal motility and enhance gut barrier function [21], while PEA exhibits anti-inflammatory properties in gastrointestinal disorders [22]. In addition to endocannabinoids and lipid mediators, certain plant-derived cannabinoids and dietary lipids can influence eCBome. For instance, fatty acids from foods such as fish, nuts, and seeds serve as precursors for endocannabinoid synthesis, while phytocannabinoids (active constituents of cannabis plant) can modulate eCBome through different receptors. We have reported the use of cannabis and different phytocannabinoids such as tetrahydrocannabinol (THC), cannabidiol (CBD), tetrahydrocannabivarin (THCV), and cannabigerol (CBG) in modulation of IBD in our previous papers [23,24,25].

eCBome is highly dynamic and responds to various external and internal stimuli. Factors such as stress, infections, inflammation, and dietary intake can influence endocannabinoid tone and signaling, thereby affecting physiological and immunological functions. For example, acute stress can transiently elevate endocannabinoid levels to restore homeostasis, while chronic stress may lead to dysregulation, contributing to pathological conditions. Disruptions in eCBome signaling have been increasingly implicated in the pathogenesis of IBD, highlighting its potential as a novel therapeutic target [26,27]. This review explores the role of eCBome in managing both intestinal and EIMs of IBD.

## 2. Extraintestinal Complications of IBD: A Role for eCBome

**Figure 1:**
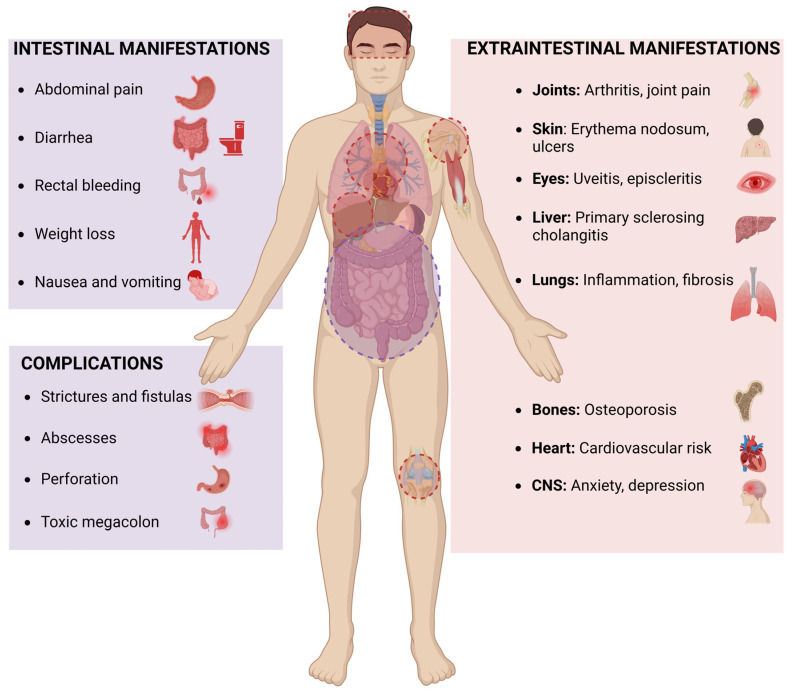
Intestinal and extraintestinal manifestations of inflammatory bowel disease.

Current therapies for IBD mainly target intestinal symptoms but often fail to manage its EIMs. Extraintestinal manifestations of IBD such as arthritis, uveitis, hepatobiliary disorders, and central nervous system disorders such as anxiety and depressions significantly impact morbidity. Extraintestinal manifestations often present alongside gastrointestinal symptoms but can also arise independently, adding complexity to disease management. Figure 1 provides a summary of the intestinal and extraintestinal manifestations of IBD. While current therapies mainly target GI-related disorders, eCBome could offer a novel therapeutic target for managing intestinal as well as EIMs. Unlike conventional therapies that predominantly focus on intestinal inflammation, eCBome modulation offers a more comprehensive approach by addressing both local and systemic manifestations of the disease. Therefore, targeting eCBome has the potential to not only reduce intestinal inflammation but also mitigate EIMs such as arthritis, metabolic dysfunction, and neurological complications associated with IBD. This broader therapeutic impact underscores translational potential. Table 1 outlines the various types of EIMs associated with IBD and highlights the potential benefits of eCBome modulation in their management.

## 3. Current Therapies in IBD: Challenges and Limitations

Despite advancements in IBD treatment, current therapies have several limitations, including incomplete response rates, loss of efficacy over time, significant adverse effects, and a failure to address EIMs of IBD, as discussed below.

### 3.1. Limited Long-Term Efficacy and Loss of Response

Many patients initially respond to conventional treatments such as aminosalicylates (e.g., mesalamine, sulfasalazine), corticosteroids (e.g., prednisone, budesonide), immunomodulators (e.g., azathioprine, methotrexate, cyclosporine), and biologics (e.g., TNF inhibitors like infliximab and adalimumab). However, up to 40% of patients experience primary non-response, and an additional 30–50% lose response over time, requiring dose escalation or switching to alternative therapies [114,115].

### 3.2. Adverse Effects and Safety Concerns

Corticosteroids, though effective for short-term symptom control, are associated with serious long-term complications, including osteoporosis, hyperglycemia, hypertension, and increased infection risk [103,104]. Immunosuppressants such as azathioprine, 6-mercaptopurine, methotrexate, cyclosporine carry risks of hepatotoxicity, myelosuppression, pancreatitis, and opportunistic infections [116,117,118]. Small-molecule inhibitors (tofacitinib) and biologic therapies such as anti-TNF, anti-integrin, and anti-IL-12/23 can increase susceptibility to infections (e.g., tuberculosis, opportunistic fungal infections) and, in rare cases, lead to the development of lymphomas or demyelinating disorders [114,119].

### 3.3. High Treatment Costs and Accessibility Issues

Biologic therapies and small-molecule inhibitors are expensive, limiting access in many regions of the world. The high cost burden can lead to treatment discontinuation, inadequate disease control, and increased hospitalization rates [114,119].

### 3.4. Surgical Interventions

Due to limitations with medical treatments and disease complications, surgical interventions remain a common intervention for 30–50% of Crohn’s disease patients, while 20–30% of ulcerative colitis patients undergo colectomy. However, infection and post-surgical recurrence in Crohn’s disease remains a significant challenge, often requiring lifelong medical therapy [120,121,122].

### 3.5. Extraintestinal Manifestations and Unmet Needs

Up to 47% of IBD patients experience extraintestinal manifestations (EIMs) affecting the joints, skin, liver, and eyes, along with symptoms such as fatigue, mental health disorders (including anxiety and depression), and dysbiosis—all of which significantly impair quality of life. Notably, EIMs can develop in up to 24% of patients before the onset of intestinal symptoms, underscoring the importance of early recognition for timely diagnosis and intervention [10,123,124]. These multifaceted complications highlight the need for novel, holistic therapeutic strategies that extend beyond conventional treatments. Current pharmacological interventions, including aminosalicylates, corticosteroids, immunosuppressants, and biologics, predominantly target inflammation within the gastrointestinal tract but often fail to address the EIMs, including systemic immune and metabolic dysregulation, associated with IBD. In contrast, eCBome modulation has demonstrated promise in regulating systemic inflammation, improving metabolic homeostasis, and restoring gut barrier integrity, making it a unique and promising therapeutic avenue. An overview of the current therapeutic landscape in IBD management, their mechanism of action, and the complexities associated with each treatment modality are highlighted below (Table 2).

In summary, current pharmacological interventions, including aminosalicylates, corticosteroids, immunosuppressants, and biologics, primarily target inflammation within the gastrointestinal tract but fail to comprehensively manage the systemic immune and metabolic dysregulation observed in IBD [135]. For example, biologics such as TNF-α inhibitors have shown limited effectiveness in addressing metabolic alterations, including insulin resistance and lipid dysregulation, which are frequently observed in IBD patients. Furthermore, these treatments do not significantly impact the gut–brain axis, which is increasingly recognized as a key player in IBD pathology. In contrast, eCBome modulation has demonstrated promise in regulating systemic inflammation, improving metabolic homeostasis, and restoring gut barrier integrity, making it a unique and promising therapeutic avenue [13,26].

## 4. The Endocannabinoidome: Components and Functions

The discovery of tetrahydrocannabinol (THC), the primary psychoactive compound in cannabis, led to the identification of the endocannabinoid system (ECS) in the early 1990s. Initially, the ECS was defined as an endogenous lipid signaling system composed of three primary components: (1) cannabinoid receptors (CB1 and CB2), (2) endogenous ligands, AEA and 2-AG, and (3) metabolic enzymes involved in their synthesis (NAPE-PLD, DAGL) and degradation (FAAH, MAGL) [14].

The ECS was originally associated with neurotransmission, pain modulation, appetite regulation, and immune responses. However, as research advanced, it became evident that additional lipid mediators, receptors, and metabolic enzymes interact with these classical components. This led to the recognition of a broader signaling network, termed the endocannabinoidome [136]. eCBome includes lipid-derived mediators synthesized from membrane phospholipids through enzymatic pathways similar to those producing classical endocannabinoids like AEA and 2-AG. To be classified within this system, these molecules must interact with cannabinoid receptors (CB1 and CB2) or functionally related receptors such as PPARs (peroxisome proliferator-activated receptors), TRPV1 (transient receptor potential vanilloid 1), and G-protein coupled receptors (GPR35, GPR55, GPR119). They must also undergo metabolic processes regulated by enzymes such as NAPE-PLD, DAGL, FAAH, and MAGL and influence physiological processes, including inflammation, metabolism, pain modulation, and gut barrier integrity [13,23,27]

**Figure 2:**
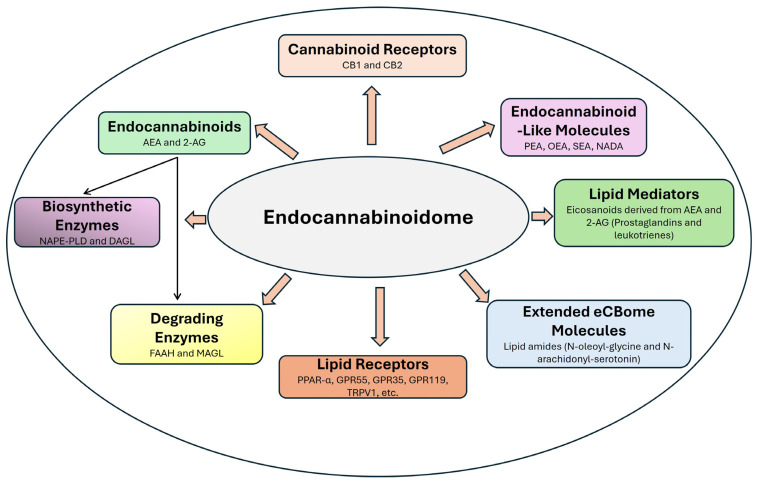
Components of endocannabinoidome. CB1—cannabinoid 1; CB2—cannabinoid 2; AEA—anandamide; 2-AG—2 arachidonylglycerol; NAPE-PLD—N-Acyl-phosphatidylethanolamine phospholipase D; DAGL—diacylglycerol lipase; FAAH—fatty acid amide hydrolase; MAGL—monoacylglycerol lipase; PPAR-α—peroxisome proliferator-activated receptor-alpha; GPR55—G protein coupled receptor 55; GPR35—G protein coupled receptor 55; GPR119—G protein coupled receptor 55; TRPV1—transient receptor potential vanilloid 1; PEA—palmitoylethanolamide; OEA—oleoylethanolamide; SEA—Stearoylethanolamide; NADA—N-arachidonoyl dopamine.

For example, although compounds like oleoylethanolamide and palmitoylethanolamide do not bind to CB1 or CB2 receptors, they activate PPARα to regulate lipid metabolism, inflammation, and energy homeostasis [137]. Similarly, TRPV1 is activated by AEA and other bioactive lipids, regulating nociception and inflammatory pathways [138]. Figure 2 provides a visual summary of the inclusion criteria and boundaries of eCBome, illustrating how its components interconnect to modulate key physiological responses.

This expanded conceptualization of the ECS—eCBome—offers new insights into therapeutic targets for various pathological conditions, including inflammatory bowel disease. Dysregulation of eCBome was implicated in IBD pathogenesis, affecting epithelial barrier integrity, cytokine production, and immune cell activation. A comprehensive evaluation of eCBome alterations in IBD is performed through biochemical, molecular, and functional assays such as lipidomic profiling (using LC-MS/MS), qPCR, Western blotting, immunohistochemistry, and receptor binding studies [16]. One key approach is lipidomic profiling, which quantifies endocannabinoids (e.g., AEA, 2-AG) and related lipid mediators (e.g., OEA, PEA) in tissues, blood, or stool samples from IBD patients using high-resolution liquid chromatography–tandem mass spectrometry (LC-MS/MS). Additionally, qPCR, Western blotting, and immunohistochemistry can evaluate gene and protein expression of key eCBome components, including CB1, CB2, PPARα, and TRPV1, and metabolic enzymes (FAAH, MAGL, DAGL, NAPE-PLD). Functional assays, such as receptor binding studies and downstream signaling assessments (e.g., ERK or AKT phosphorylation), provide further insight into the functional consequences of eCBome dysregulation. Integrating these approaches with clinical markers, histopathological findings, and disease severity assessments enhances our understanding of eCBome alterations in IBD [14,20]. Studies have reported elevated levels of AEA and 2-AG, reduced CB1/CB2 expression, and altered activity of metabolic enzymes in inflamed colonic tissues, supporting the role of eCBome dysregulation in IBD [16,139,140]. Additionally, lower levels of PEA and OEA in IBD further highlight the importance of these non-canonical mediators and underscore the therapeutic potential of targeting eCBome [141]. Dietary interventions enriched with PEA and OEA have shown therapeutic benefits in IBD [21,22]. Overall, this historical background and definition establish clear boundaries for eCBome, providing a framework for understanding the inclusion of molecules such as PPARα and TRPV1, and underscoring their relevance as potential therapeutic targets in IBD.

A range of ethnomedicinal, preclinical, and clinical studies suggest that targeting eCBome with phytocannabinoids (e.g., cannabidiol, THC), synthetic cannabinoids (e.g., HU308), or enzyme inhibitors (e.g., JZL195) may reduce colonic inflammation, alleviate symptoms, and promote mucosal healing [23,24,25,139,142]. However, despite promising findings, further clinical trials are needed to optimize dosing, delivery methods, and long-term safety, positioning eCBome as a novel therapeutic target for IBD.

## 5. Metabolic Dysregulation in IBD

Metabolic disorders are among the most commonly reported EIMs of IBD, significantly impacting morbidity and overall patient health [143]. These disturbances often arise from chronic systemic inflammation, nutritional deficiencies, altered gut microbiota, and the side effects of medications. Below, we detail the key metabolic complications in IBD along with their underlying mechanisms.

### 5.1. Dyslipidemia

IBD patients frequently exhibit altered lipid profiles, characterized by decreased high-density lipoprotein (HDL) and increased triglycerides and low-density lipoproteins (LDL) [144]. Chronic inflammation in IBD elevates pro-inflammatory cytokines (e.g., IL-6, TNF-α) that disrupt normal lipid metabolism. These cytokines promote lipid peroxidation and alter hepatic lipid clearance. The imbalance in lipid processing contributes to the development of a pro-atherogenic profile, thus increasing the risk of cardiovascular diseases [145,146,147].

### 5.2. Insulin Resistance and Diabetes

Systemic inflammation and corticosteroid use in IBD can impair glucose metabolism, leading to insulin resistance and an increased risk of diabetes [148]. Pro-inflammatory cytokines such as TNF-α interfere with insulin receptor signaling by promoting serine phosphorylation of insulin receptor substrates, thereby reducing insulin sensitivity. Additionally, corticosteroids enhance gluconeogenesis, further exacerbating insulin resistance. This creates a vicious cycle where impaired insulin signaling contributes to sustained inflammation and metabolic dysregulation [149].

### 5.3. Bone Metabolism Abnormalities

Osteoporosis and osteopenia are common in IBD, largely due to malabsorption of calcium and vitamin D, chronic inflammation, and prolonged corticosteroid use [150]. Inflammatory cytokines, notably IL-6 and TNF-α, stimulate osteoclast differentiation and activation, leading to increased bone resorption and reduced bone mineral density. Corticosteroids further impair osteoblast function and contribute to calcium loss, collectively increasing the risk of fractures and compromising skeletal integrity [151].

### 5.4. Body Composition Changes

Conditions such as cachexia and sarcopenia are frequently observed in IBD patients. Additionally, fat redistribution—especially central obesity—can negatively impact functional capacity and disease prognosis [73,152]. Systemic inflammation, driven by cytokines like TNF-α, IL-1, and IL-6, accelerates muscle protein degradation while inhibiting muscle protein synthesis, leading to sarcopenia and cachexia. Hormonal imbalances and adipose tissue inflammation further contribute to the redistribution of fat mass, often resulting in central obesity despite overall weight loss.

### 5.5. Gut Microbiota and Metabolic Alterations

Gut microbiota and metabolomic alterations play a crucial role in IBD-related metabolic disturbances [153]. Dysbiosis, characterized by reduced microbial diversity and altered metabolomic profiles, is a hallmark of IBD and plays a crucial role in metabolic disturbances [154]. Alterations in the gut microbiota led to decreased production of beneficial short-chain fatty acids (SCFAs), such as butyrate, which normally have anti-inflammatory effects and support energy homeostasis. Disruption in bile acid metabolism also contributes to impaired lipid absorption and energy regulation, further exacerbating systemic metabolic imbalances [21,155].

## 6. Modulatory Role of eCBome in Metabolic Dysregulation

eCBome plays a pivotal role in regulating energy balance, lipid metabolism, insulin sensitivity, and gut–brain signaling. Its dysregulation has been linked to obesity, type 2 diabetes, and metabolic syndrome—conditions that frequently co-exist with IBD and worsen disease outcomes [15,27,153,156]. Endocannabinoids such as anandamide and 2-arachidonoylglycerol signal through cannabinoid receptors (CB1 and CB2), as well as TRP channels and PPARs. In IBD, these molecules are dysregulated in both the intestinal mucosa and systemic circulation [11,112,157,158]. Activation of CB2 receptors on immune by endocannabinoids reduces cytokine production and inhibits macrophage activation, thereby mitigating inflammation [159,160]. CB1 receptor activity in adipose tissue and liver regulates lipid storage and oxidation. Targeting peripheral CB1 receptors may help ameliorate dyslipidemia while avoiding central side effects [161,162,163]. Through interactions with CB1 and PPARγ pathways, endocannabinoids regulate insulin sensitivity and glucose uptake. Inhibition of CB1 receptors has been shown to improve insulin resistance in IBD [153,164,165]. CB2 receptor activation is reported to inhibit osteoclastogenesis, promoting bone preservation. This pathway offers therapeutic potential for IBD-associated osteoporosis [166]. Altered gut microbiota is shown to affect eCBome tone, affecting metabolite production and intestinal barrier integrity. Restoring balance in this axis may help mitigate metabolic dysregulation [153]. Endocannabinoid-like molecules such as OEA and PEA further support metabolic regulation. OEA promotes GLP-1 release, enhances fat oxidation, and reduces food intake—primarily through GPR119—while PEA exerts anti-inflammatory and gut-protective effects [21,22,79,141,167]. In high-fat diet-induced obese mice, treatment with synthetic GPR119 agonists such as ps297 and ps3188, in combination with sitagliptin—a DPP-IV inhibitor—has been shown to retard body weight gain. This combination therapy also improved glycaemic control and enhanced hepatic health, as evidenced by improved GLP-1 regulation, better liver histopathological scores, and modulation of metabolic hormones [79,80]. In addition to endogenously produced endocannabinoids and endocannabinoid-like molecules, several exogenous compounds including diet, synthetic and phytocannabinoids play a pivotal role in regulation of metabolic processes and pathogenesis of metabolic dysregulation as discussed previously [13,168,169,170]

Together, these insights underscore the critical role of metabolic dysregulation in IBD pathogenesis and highlight the potential of eCBome-targeted therapies to address these complex systemic disturbances.

## 7. Role of GLP-1 and eCBome in Metabolic Dysregulation in IBD

GLP-1 is an incretin hormone secreted by intestinal L-cells in response to food intake and plays a pivotal role in glucose homeostasis, lipid metabolism, and energy regulation [171,172,173]. Despite its influence on metabolism and inflammation, GLP-1 is not considered part of eCBome because eCBome is defined as a network of lipid-derived mediators, receptors, and metabolic enzymes that interact with cannabinoid receptors (CB1, CB2) or related receptors (e.g., PPARs, TRPV1, GPR55) [136,174]. Instead, GLP-1 is a peptide hormone derived from proglucagon and acts on GLP-1 receptor (GLP-1R), a GPCR unrelated to ECS signaling [172]. Furthermore, the biosynthesis and degradation pathways differ markedly between these systems. Endocannabinoidome molecules are synthesized from membrane phospholipids and are metabolized by enzymes such as FAAH and MAGL, whereas GLP-1 is produced by intestinal L-cells and rapidly degraded by DPP-IV [175]. Although GLP-1 signaling modulates ECS activity, such as reducing CB1 overactivation, enhancing CB2-mediated anti-inflammatory effects, and modulating gut microbiota, it only exhibits crosstalk with the ECS rather than being a component of eCBome [176,177].

Growing evidence suggests a complex interplay between GLP-1 and eCBome in regulating metabolism, gut homeostasis, and inflammation, making them promising therapeutic targets for IBD-associated metabolic disorders [173,178,179,180]. Dual modulation of GLP-1 and ECS, for example, combining GLP-1 receptor agonists (such as liraglutide or semaglutide) with CB1 antagonists may enhance insulin sensitivity, reduce fat accumulation, and control inflammation. Additionally, GLP-1 analogs help restore gut barrier integrity, complementing the ECS’s function in regulating intestinal permeability and inflammation. Additionally, CB2 receptor activation, using selective agonists like HU308, could complement GLP-1 therapy by reducing systemic inflammation, metabolic dysregulation and maintaining gut homeostasis [24]. Furthermore, endocannabinoid-like molecules such as PEA and OEA are shown to enhance GLP-1 potency, providing further metabolic benefits and improving overall therapeutic outcomes [181]. Table 3 summarizes key studies on the interaction between GLP-1 and eCBome in metabolic dysregulation in IBD.

In summary, the interplay between GLP-1 and eCBome plays a crucial role in regulating metabolic dysregulation in IBD. Targeting this axis may not only alleviate metabolic complications but also mitigate inflammation and promote gut health, offering a promising therapeutic approach for IBD management. Further clinical studies are needed to explore the synergistic effects of GLP-1 and ECS-targeted therapies in treating metabolic disorders associated with IBD.

## 8. Beyond the Gut: Future Perspectives on eCBome Modulation in IBD

The complexity of IBD extends far beyond gut inflammation, with extraintestinal manifestations significantly contributing to disease burden and reducing patients’ quality of life. Conventional therapies primarily focus on intestinal inflammation, often overlooking the systemic effects of IBD. The endocannabinoidome, an intricate network of lipid mediators, receptors, and enzymes, has emerged as a promising target for modulating both intestinal and extraintestinal complications of IBD. Recent evidence suggests that eCBome regulation influences key physiological processes, including immune responses, metabolism, and neuroinflammation, positioning it as a potential therapeutic avenue for holistic disease management. CB1 and CB2 receptors, two key components of eCBome, are shown to be highly dysregulated in IBD. Use of selective/peripheral CB1 antagonists and CB2 agonists could be employed as a promising drug candidate for addressing specific metabolic disturbances without central side effects. Our previous studies using DSS-induced acute and chronic colitis murine models demonstrated that activation of the CB2 receptor with the selective agonist HU308, and activation of the CB1 receptor using a sub-therapeutic dose of THC in combination with the CB1 allosteric modulator ZCZ011 significantly improved both colonic and systemic markers of IBD [24,25]. These included reductions in diarrhea, rectal bleeding, body weight loss, colon histopathological scores, organ dysfunction (liver, kidney, and spleen), and pain-related behaviors.

Most importantly, both treatments restored systemic GLP-1 and ammonia levels to those observed in healthy controls, underscoring their potential role in mitigating systemic complications of IBD. Elevated systemic ammonia levels have been linked to central nervous system (CNS) disorders, including Alzheimer’s disease. Notably, IBD patients frequently experience CNS-related symptoms such as anxiety and depression, which may be influenced by ammonia dysregulation [190,191]. Investigating the role of ammonia regulation in IBD patients could provide valuable insights into the gut–brain axis and its implications for disease management.

Diet and nutrition also play a significant role in etiopathogenesis of IBD. While triggers may vary from person to person, a Western-style diet rich in processed foods, unhealthy fats (trans fats), sugars, and additives generally worsens IBD [192]. Moreover, diet is pivotal in modulating both the gut microbiome and gut–brain axis. Unhealthy dietary patterns often linked to microbial dysbiosis; a condition characterized by an altered balance in the gut microbiota. This dysbiosis is well-documented in IBD and is associated with increased intestinal permeability, immune dysregulation, and chronic dietary interventions rich in fiber, polyphenols, and omega-3 fatty acids have shown promise in modulating the gut microbiota and reducing inflammation [22,193]. For example, beta-caryophyllene, a terpene abundant in cannabis and also considered a dietary cannabinoid, has been demonstrated to reduce DSS-induced colitis in mice through CB2 and PPAR-α activation [194,195] and has also shown beneficial effects in diabetes [185,186].

Probiotic supplementation has gained attention as an adjunct therapy for colitis due to its ability to restore microbial balance and enhance intestinal barrier integrity. Clinical studies suggest that probiotics such as *Escherichia coli* Nissle 1917 and multi-strain formulations like VSL#3 can induce remission and maintain mucosal healing in IBD patients [196,197]. Mechanistically, probiotics modulate the immune response by increasing short-chain fatty acid production, promoting regulatory T cell responses, and suppressing pro-inflammatory cytokine secretion [198,199]. Additionally, through modulation of microbiome, probiotics can also positively influence eCBome tone in IBD [200]. Integrating cannabinoid-targeted therapies with existing dietary, anti-inflammatory, and metabolic interventions could enhance therapeutic outcomes.

However, while promising, the efficacy of probiotic remains strain-dependent, and further clinical trials are needed to establish optimal formulations and long-term benefits. A personalized probiotic regimen, tailored to individual patient profiles and combined with standard pharmacotherapy, may offer significant benefits in managing both the intestinal and EIMs of IBD. Furthermore, emerging evidence suggests that other components of eCBome, such as GPR35 and GPR55, are dysregulated in IBD. The activation of GPR35 by olsalazine, an aminosalicylate used in IBD therapy, appears to play a role in preventing colitis [201,202].

While eCBome represents a promising therapeutic avenue for IBD, Table 4 outlines key challenges in its clinical translation and proposes potential solutions for future research.

## 9. Conclusions

Extraintestinal manifestations of IBD pose a significant and diverse array of clinical challenges, significantly impacting patients’ lives and healthcare utilization. While conventional therapies primarily target gut inflammation, the endocannabinoidome emerges as a promising and versatile target for managing inflammatory, metabolic, and extraintestinal complications of IBD. Preliminary evidence highlights its therapeutic potential, but further research is essential to optimize clinical applications and ensure safety.

A multidisciplinary approach is crucial to delivering holistic care, addressing not only gut inflammation but also improving overall quality of life and reducing the burden of EIMs. Mechanistic investigations of eCBome signaling at the cellular level, particularly its downstream pathways, such as MAPK/ERK and PI3K/AKT, could provide deeper insights for designing effective targeted therapies. By addressing these unmet needs, eCBome modulation has the potential to transform IBD management and significantly enhance patient outcomes.

## Figures and Tables

**Table 1:** Benefits role of endocannabinoidome modulation in extraintestinal manifestations of IBD.

Category	Extraintestinal Manifestations	Beneficial Role of eCBome Modulation
Musculoskeletal conditions	Peripheral and axial arthritis, osteoporosis, and osteopenia [28,29,30,31]	Cannabis and cannabinoids are shown to reduce joint pain and inflammation [32,33,34,35], osteoporosis and osteopenia [36,37,38].
Dermatological conditions	Erythema nodosum, pyoderma gangrenosum, psoriasis [39,40,41]	Cannabinoids have demonstrated anti-inflammatory and immunomodulatory potential in skin disorders [42,43,44,45], chronic wound healing, and skin ulcers [46,47,48].
Ocular disorders	Uveitis, conjunctivitis, and episcleritis [49,50,51,52,53]	Cannabinoids reduce a multiple range of ocular disorders including uveitis, proliferative vitreoretinopathy intraocular pressure, corneal pain, and inflammation [54,55,56,57,58,59,60].
Hepatobiliary complications	Primary sclerosing cholangitis (PSC)—inflammation and narrowing of the bile ducts, fatty liver, and cirrhosis [61,62,63]	Cannabinoids have demonstrated a beneficial role in range of liver disorders including liver fibrosis, fatty liver disease, etc. [64,65,66,67,68,69].
Metabolic disorders	Metabolic syndrome (dyslipidemia, diabetes, obesity, and hypertension) [7,70,71,72,73], vitamin D and calcium deficiency [74,75], metabolic bone syndrome in children [76]	Modulation of eCBome is shown to be beneficial in various forms of metabolic disorders including obesity, diabetes, and dyslipidemia via the modulation of Glucagon like peptide-1 (GLP-1) hormone [27,77,78,79,80,81].
Renal complications	Nephrolithiasis (kidney stones), abnormal kidney profiles and renal function [82,83,84,85,86]	Cannabinoids reduce renal hypertrophy and have beneficial role in chronic kidney diseases [24,87,88].
Hematological complications	Anemia (iron-deficiency and anemia of chronic disease), thrombophlebitis, leukocytosis [89,90]	eCBome modulation has a beneficial role in blood cell development, immune disorders, and hematological malignancies [91,92].
Neurological symptoms	Dementia, depression, anxiety, Alzheimer’s disease, peripheral neuropathy, multiple sclerosis [8,93,94]	Cannabinoids reduce various forms of neurological disorders including neuropathic pain, multiple sclerosis, depression and anxiety, etc. [95,96,97,98,99].
Endocrine	Thyroid disorders [100,101], adrenal insufficiency associated with long-term corticosteroid use [102,103,104]	Cannabidiol is shown to improve thyroid function via modulating vitamin D3 receptor [105]. Increasing evidence of cannabinoids in endocrine disorders [106,107].
Cardiovascular	Increased risk of cardiovascular disease [108,109,110]	eCBome modulation is beneficial in improving cardiovascular functions in range of pathophysiology such as hypertension, sepsis, atherosclerosis, etc. [111,112,113].

**Table 2:** Current IBD therapies and challenges.

Therapies	Mechanism of Action	Limitation	Complications/Side Effects
Aminosalicylates	Inhibits cyclooxygenase and lipoxygenase pathways to reduce prostaglandins and leukotrienes, decreasing inflammation.	Limited efficacy in Crohn’s disease.	Headache, nausea, and, rarely, kidney dysfunction, adherence issues [118,125].
Corticosteroids	Activates glucocorticoid receptors to suppress inflammatory gene transcription, reducing cytokine production and immune cell activity.	Effective for short-term flare management but not suitable for long-term use.	Weight gain, osteoporosis, hypertension, and increased infection risk, risk of dependency and withdrawal challenges [103,104].
Immunomodulators	Inhibits DNA synthesis and T-cell proliferation, reducing immune system overactivity.	Delayed onset of action (may take months), regular monitoring required.	Increased susceptibility to infections, potential liver toxicity and bone marrow suppression [116,117,118].
Biologic therapies	Targets specific immune mediators (e.g., TNF-alpha, integrins, or interleukins) to reduce inflammation and immune cell migration.	Risk of infusion/injection site reactions, development of antibodies reducing efficacy, high cost, and limited accessibility.	Long-term safety concerns, including malignancy risk [114,119].
Small-molecule inhibitors	Blocks intracellular signaling pathways (e.g., Janus kinase pathways) or prevent lymphocyte migration to reduce inflammation.	Limited long-term safety/efficacy data.	Potential off-target effects, monitoring for adverse reactions necessary [114,126,127].
Antibiotics	Modifies gut microbiota by reducing bacterial overgrowth and addressing infections or abscesses.	Limited efficacy; primarily used for specific complications.	Risk of altering gut microbiota adversely, potential development of antibiotic resistance, risk of flare-ups [128,129].
Probiotics	Modulates gut microbiota to restore balance and reduce inflammation by promoting beneficial bacterial strains.	Variable efficacy, lack of standardized formulations and dosing guidelines.	Uncertainty regarding long-term benefits [130,131].
Fecal microbiota transplant	Restores gut microbial diversity and function, potentially reducing inflammation and promoting remission.	Emerging therapy with variable success rates and regulatory and ethical considerations.	Concerns about long-term safety and standardization [132,133,134].
Surgical interventions	Removes diseased portions of the intestine to reduce inflammation and improve symptoms.	Risks associated with surgery: infection, anesthesia complications.	Possibility of disease recurrence post-surgery, impact on quality of life, and potential need for ostomy [120,121,122].

**Table 3:** Interaction between glucagonlike peptide-1 and the endocannabinoidome in metabolic dysregulation in inflammatory bowel disease.

eCBome Modulation	Mechanism of Action	Potential Benefits in IBD-Associated Metabolic Dysregulation	References
CB1 receptor agonist	CB1 agonist in combination with CB1 receptor allosteric modulator normalized systemic and colonic GLP-1 levels.	Restores GI homeostasis and body weight.	[24,25]
GLP-1 receptor agonist	GLP-1 improves gut barrier integrity and reduces metabolic inflammation.	GLP-1 agonists reduce IBD-associated metabolic complications.	[173,178,182]
CB1 antagonist	CB1 blockade reduces body weight, insulin resistance and metabolic inflammation.	The combination of peripheral CB1 antagonist and GLP-1 receptor (GLP-1R) agonist reduce body weight, fat mass and metabolic syndrome in IBD.	[177,183]
Endocannabinoid-like molecules (OEA, PEA)	OEA and PEA regulate satiety, gut permeability, and lipid metabolism.	Enhances GLP-1 release, reduces food intake and improves gut-barrier integrity.	[22,141,184]
CB2 receptor agonist	CB2 activation reduces metabolic inflammation, insulin resistance and enhances immune regulation.	Reduces osteoporosis risk and improves insulin sensitivity. CB2 agonists combined with GLP-1R agonists could provide dual metabolic and anti-inflammatory benefits.	[169,185,186,187]
eCBome-GLP-1 crosstalk	Enhances GLP-1 secretion while reducing CB1-driven metabolic dysfunction.	Highlights the need for eCBome-GLP-1 targeted interventions in metabolic disorders of IBD.	[27]
Diets and probiotics	Increases GLP-1 resulting in decreased glucose and insulin resistance	Beneficial in managing EIMs of IBD including diabetes.	[188,189]

**Table 4:** Challenges and potential solutions for modulating endocannabinoidome in inflammatory bowel disease.

Risks Category	Challenges	Potential Therapeutic Strategies
Psychoactive side effects	Activation of CB1 receptors, especially by brain-penetrant cannabinoids, carries the risk of psychoactive and behavioral side effects [203,204,205].	Selective modulators: Use of non-psychoactive cannabinoids like CBD, peripherally restricted cannabinoids, and selective modulators targeting specific eCBome pathways can bypass CB1-mediated side effects [24,161]. Allosteric modulators: Use of CB1 receptor allosteric modulators and targeted delivery systems can enhance therapeutic precision while minimizing psychoactive side effects [25].
Immune suppression	Prolonged CB2 receptor engagement could lead to systemic immune suppression, potentially altering immune homeostasis [160,206].	Selective targeting: Developing tissue-specific CB2 receptor modulators can help minimize systemic immune suppression. For example, targeting CB2 in the gut to modulate localized inflammation while avoiding systemic immune effects. Short-term or intermittent dosing: Intermittent dosing schedules could help maintain immune homeostasis. Combination therapies: Combining sub-therapeutics doses of CB2 agonist and conventional treatments such as biologics, immunosuppressant, 5 aminosalicylates, or microbiome-targeted therapies may yield synergistic benefits and enhance efficacy and safety.
Off-target effects	eCBome involves non-cannabinoid receptors, such as TRPV1 and GPR55, increasing the likelihood of off-target effects [174].	Development of highly selective ligands: The design and use of molecules that selectively target specific cannabinoid receptors (e.g., CB2) while avoiding the activation of non-cannabinoid receptors like TRPV1 or GPR55 can overcome off-target side effects.
Variability in responses	Genetic, metabolic, and microbiome differences among individuals lead to varied responses to cannabinoids, complicating treatment optimization.	Personalized medicine: Stratifying patients based on genetic, epigenetic, and microbiome profiles could optimize eCBome-targeted treatments.

## Data Availability

Not applicable.

## Abbreviations

The following abbreviations are used in this manuscript:

2-AG	2-Arachidonoylglycerol
AEA	Anandamide
ASA	Aminosalicylates
CB1	Cannabinoid receptor 1
CB2	Cannabinoid receptor 2
CBD	Cannabidiol
DAGL	Diacylglycerol lipase
ECS	Endocannabinoid system
eCBome	Endocannabinoidome
EIMs	Extraintestinal manifestations
FAAH	Fatty acid amide hydrolase
GLP-1	Glucagon-Like Peptide-1
GLP-1R	Glucagon-Like Peptide-1 Receptor
GPR35	G Protein-Coupled Receptor 35
GPR55	G Protein-Coupled Receptor 55
GPR119	G Protein-Coupled Receptor 119
HDL	High-density lipoprotein
IBD	Inflammatory bowel disease
IL-6	Interleukin-6
IL-12	Interleukin-12
IL-23	Interleukin-23
JAK	Janus kinase
LDL	Low-density lipoprotein
MAGL	Monoacylglycerol lipase
NADA	N-arachidonoyl dopamine
NAPE-PLD	N-Acyl-phosphatidylethanolamine phospholipase D
OEA	Oleoylethanolamide
PEA	Palmitoylethanolamide
PPAR-α	Peroxisome proliferator-activated receptor alpha
SCFAs	Short-chain fatty acids
SEA	Stearoylethanolamide
THC	Tetrahydrocannabinol
TNF-α	Tumor Necrosis Factor-Alpha
TRPV1	Transient receptor potential vanilloid 1
